# MiRNAs as major players in brain health and disease: current knowledge and future perspectives

**DOI:** 10.1038/s41420-024-02283-x

**Published:** 2025-01-13

**Authors:** Sarika V. Kapplingattu, Sujata Bhattacharya, Yogita K. Adlakha

**Affiliations:** Amity Institute of Molecular Medicine and Stem Cell Research, Amity University, Noida, Uttar Pradesh 201303, India

**Keywords:** Cancer in the nervous system, miRNAs, Neurodegeneration

## Abstract

MicroRNAs are regulators of gene expression and their dysregulation can lead to various diseases. MicroRNA-135 (MiR-135) exhibits brain-specific expression, and performs various functions such as neuronal morphology, neural induction, and synaptic function in the human brain. Dysfunction of miR-135 has been reported in brain tumors, and neurodegenerative and neurodevelopmental disorders. Several reports show downregulation of miR-135 in glioblastoma, indicating its tumor suppressor role in the pathogenesis of brain tumors. In this review, by performing in silico analysis of molecular targets of miR-135, we reveal the significant pathways and processes modulated by miR-135. We summarize the biological significance, roles, and signaling pathways of miRNAs in general, with a focus on miR-135 in different neurological diseases including brain tumors, and neurodegenerative and neurodevelopmental disorders. We also discuss methods, limitations, and potential of glioblastoma organoids in recapitulating disease initiation and progression. We highlight the promising therapeutic potential of miRNAs as antitumor agents for aggressive human brain tumors including glioblastoma.

## Facts

- MicroRNAs play significant regulatory roles in brain tumors and neurological diseases.
- Brain-specific miR-135 regulates several signaling pathways, such as Hippo and Insulin pathways in brain tumors and neurological diseases.
- A comparative evaluation of different methods for generating glioblastoma organoids, a model to study the tumorigenesis, drug resistance, and therapeutic development.
- MiRNAs, including miR-135, display significant therapeutic potential for treating brain disorders.

## Open questions

- How is miR-135 regulated at the epigenetic and post-transcriptional levels in cancer?
- What is the role of alternative splicing and cell-type-specific transcription factors in modulating the expression of miR-135 in different cancers?
- Does modulating miR-135 expression have therapeutic and diagnostic applications in brain tumors and neurological disorders?

## Introduction

MicroRNAs (miRNAs) represent a large group of endogenous non-coding RNAs, approximately 19–25 nucleotides in length, that regulate gene expression in plants and animals either through the degradation of mRNA or the suppression of protein synthesis [1]. MiRNAs account for 1–5% of the human genome and refine the expression of nearly 30% of genes [2]. They regulate a variety of biological processes, including proliferation, apoptosis, development, and immune response [3]. Given their important biological roles, any dysregulation in their biogenesis or function can contribute to various diseases, including cancer and neurological diseases. Several miRNAs have been shown to influence the hallmarks of cancer [4].

Brain tumors are among the most lethal types of tumors found in both children and adults wherein single gene mutations and radiation are primary risk factors. Brain tumors can be both malignant and non-malignant [5]. Other than tumors, neurological diseases such as neurodegenerative and neurodevelopmental diseases also affect the central and peripheral nervous system in an acute or chronic manner. Neurodegenerative disorders exhibit irreversible damage and death of neurons caused by mutated genes or pathologically altered proteins. They are more commonly associated with old age and share the common symptoms of dementia [6]. Neurodevelopmental disorders are broadly defined as behavioral and cognitive disorders that are characterized by significant challenges in learning, acquiring, and implementing specific social, motor, or intellectual brain functions [7]. Moreover, the pathophysiology of these diseases remains unclear, and as a result, no effective treatment or cure is available, making them more harmful [6].

MiRNAs-mediated regulation of various pathways in these brain diseases is of paramount significance with respect to therapeutic development. Therefore, in this review, we summarize the biological significance, roles, and signaling pathways of miRNAs in general, with a focus on miR-135 in different neurological diseases including brain tumors, and neurodegenerative and neurodevelopmental disorders.

## MiRNA biogenesis

MiRNA biogenesis is a two-step process with nuclear cleavage and cytoplasmic maturation in both canonical and non-canonical pathways. Most of the miRNAs are transcribed from gene sequence by RNA polymerase II/III into primary miRNAs (pri-miRNAs), which are several kilobases in length. The microprocessor complex, composed of the Drosha and DiGeorge syndrome critical region genome 8 (DGCR8) proteins, processes pri-miRNA into 60–70 nt long precursor miRNAs (pre-miRNAs) [8]. DGCR8 binds to pri-miRNA by recognizing N6-methyladenylated motifs within the pri-miRNA [9], while Drosha cleaves the hairpin loop structure of pri-miRNA duplex, generating a 2nt 3’ overhangs on pre-miRNA [10]. Pre-miRNAs are transported to cytoplasm through the nuclear pores by a protein complex comprising of Exportin-5 and RanGTP [11]. In the cytoplasm, Dicer, an RNase III endonuclease, and its associated trans-activation response RNA binding protein (TRBP) process the pre-miRNA to yield double-stranded~19–25 nt mature miRNA strands [12]. The mature miRNA strand with lower 5’ stability or 5’ uracil is selectively loaded into the Argonaute (AGO) family of proteins, becoming the guide miRNA, while the unloaded strand, known as the passenger strand, is unwound from guide miRNA and cleaved by AGO2 protein, depending upon mismatches [13]. The unwound mature guide miRNA, after being loaded into AGO protein along with cofactor GW182, forms the RNA-induced silencing complex (RISC), which binds to the 3’UTR of target mRNA to stimulate translational suppression or mRNA degradation [11, 12].

## Regulation of gene expression by MiRNA

MiRNAs regulate their target mRNAs by inducing translational repression, deadenylation, and decapping [13]. MiRNAs generally bind to complementary sequences known as miRNA response elements (MREs) in the 3’ UTR region of mRNA [14, 15]. However, some MREs have also been discovered in the 5’ UTR, coding regions, and promoter regions, where they exert silencing and inducing effects on gene expression, respectively [16–18].

Regulation of gene expression by miRNAs occurs through AGO2-dependent degradation of target mRNAs or miRISC-mediated translational inhibition followed by target mRNA decay [19]. The extent of complementarity between the seed region of the miRNA and the MREs determines the fate of the target mRNA: complete complementarity favors AGO2-dependent degradation of the target mRNA, while partial complementarity facilitates miRISC-mediated translational inhibition followed by target mRNA decay [19]. Complete complementarity between the miRNA and MRE facilitates the endonuclease action of AGO2 on the target mRNA, followed by cleavage. However, partial complementarity between the miRNA and MRE is generally observed in animals due to the presence of mismatches, which restrict AGO2 activity and favor miRISC-mediated translational inhibition [16].

In general, miRNAs regulate proteins in proliferating cells by downregulation of target mRNAs, although a few studies suggest the upregulation of target mRNA by miRNAs via direct interaction with the 3’ UTR region of target mRNA. These upregulation mechanisms require Fragile X mental retardation gene 1 (FXR1) and AGO2 protein [20].

## Dysregulation of miRNAs in cancer

A large number of miRNAs are dysregulated in several diseases, such as cancer. The genomic and epigenomic variations of miRNAs and transcription factors contribute to the alteration of miRNAs in cancers [15]. Several studies suggest that the modulation of transcription factors leads to abnormal transcription of pre-miRNAs in cancers. For instance, miR-145 is regulated by transcription factors such as p53, RREB1, β-catenin/TCF4, FOXO1, and FOXO3 in renal cancer [21]. In BRAF-mutant melanomas, the microphthalmia-associated transcription factor (MITF) positively regulates the expression of miR-579-3p, which in turn downregulates BRAF, stabilizes MITF protein, and induces its own transcription in a positive feedback regulatory loop [22–24]. Another transcription factor that directly regulates the miR-34a family is p53, which demethylates the CpG island of miR-34a and thus activates the expression of miR-34a in colorectal cancer, leading to decreased growth, metastasis, and inhibition of tumor stem cells [15, 20]. Additionally, the activity of p53 can be induced by miR-34a through the inhibition of the NAD-dependent deacetylase sirtuin-1 (SIRT1) [15, 21, 23, 24]. In gastric cancer, the zinc finger protein 52 (ZNF521) enhances proliferation, invasion, and migration by inhibiting apoptosis via regulation of miR-204-5p [25]. MiR-30 has been identified as a tumor suppressor in glioma, as overexpression of miR-30 leads to suppression of proliferation, migration, and invasion of glioma cells by regulating the expression of SOX9 [26]. In Acute Myeloid Leukemia (AML), an inverse correlation between meningioma-1 (MN1) and miR-181b and miR-20a has been observed [27]. In bladder cancer, CpG methylation inactivates miR-200 [28]. The regulation of the microprocessor NF90/NF45 inhibits the processing of pre-miR-7 in hepatocellular carcinoma [29]. Circular RNAs also regulate the expression of miRNAs in cancer, such as exosomal circSTRBP, which modulates the expression of miR-1294/miR-593-3p during the progression of gastric cancer [30]. One such miRNA is miR-135, which has shown extensive dysregulation in cancer.

## MiR-135

MiR-135a and miR-135b are members of the miR-135 family. In humans, miR-135a is encoded by the MIR135A1 and MIR135A2 genes, which are located on chromosomes 3p21.2 and 12q23.1, respectively. MiR-135b is transcribed by the MIR135B gene on chromosome 1q32.1. Although miR-135 is considered a brain-specific miRNA, its expression has been reported in various organs, including the artery, colon, breast, and lung [31]. MiR-135a is known to regulate neuronal morphology, neural induction, synaptic function, glutamatergic neurotransmission, adult neurogenesis, and neural progenitor cell proliferation [32]. The aberrant function of miR-135 has been reported in several diseases, such as Alzheimer’s disease [33], atherosclerosis [34], osteoporosis [35], pulmonary fibrosis [36], and cancer [37]. Dysregulation of miR-135 leads to its oncogenic and tumor-suppressive roles in cancer [37, 38]. MiR-135 regulates cell proliferation and invasion through various signaling pathways, such as MAPK and JAK2/STAT3 [37]. In turn, the expression of miR-135 is known to be regulated by the transcription factor PAX6 [39].

## Biological significance of miR-135

**Fig. 1:**
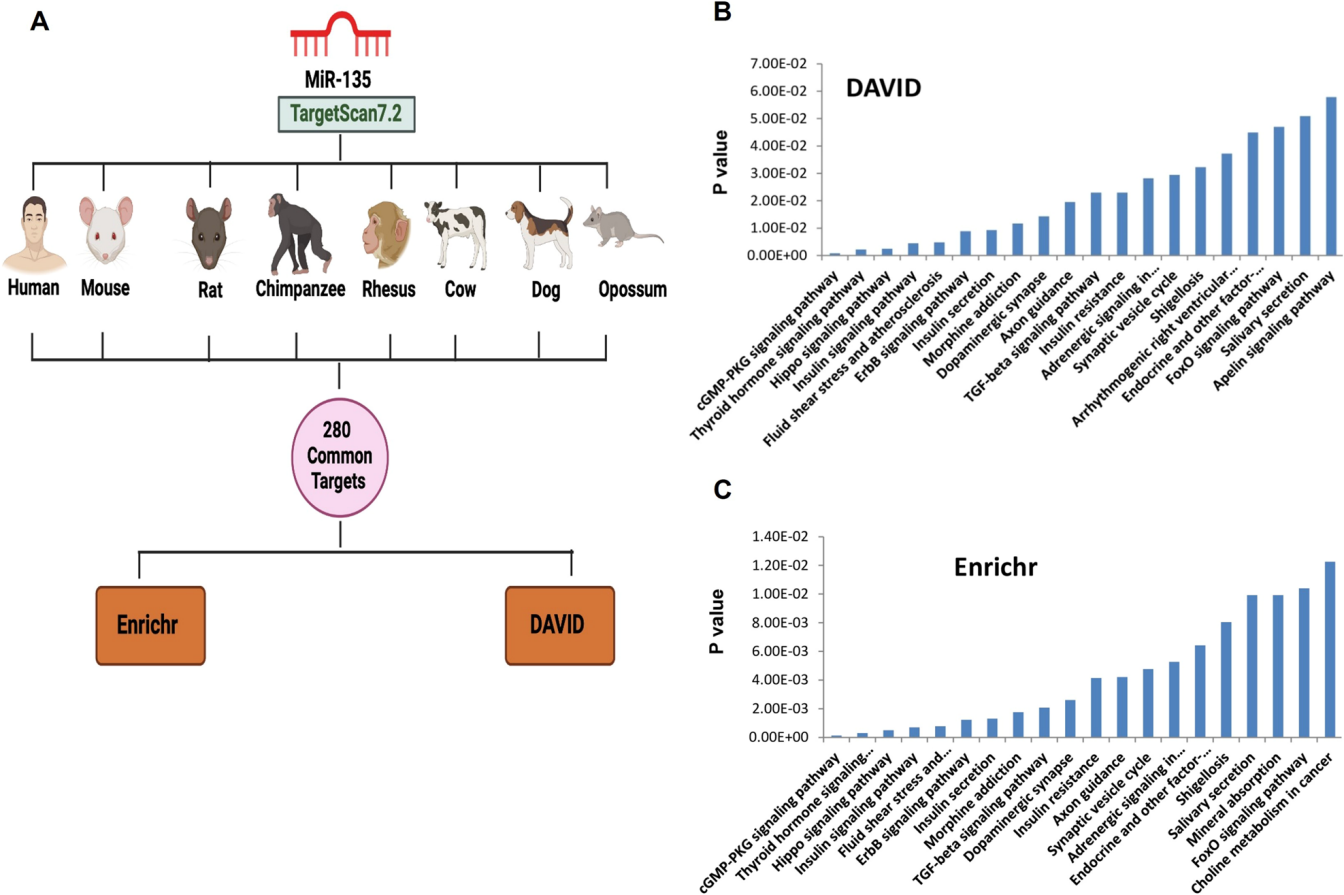
Biological significance of miR-135. (**A**) Schematic representing the method of in silico analysis: A list of predicted targets of miR-135 was prepared using the TargetScan7.2 program (43). To find evolutionary conserved functions of miR-135, we found 280 conserved predicted targets from 8 species (Human, Mouse, Rat, Chimpanzee, Rhesus, Cow, Dog, Opossum). We used these 280 conserved target genes in Enrichr and DAVID database analysis to reveal enriched pathways modulated by miR-135. (**B**-**C**) Bar diagram representation of enriched biological pathwaysusing predicted targets of miR-135 by DAVID and Enrichr database analysis.

We questioned the biological significance of miR-135. Since the targets of miRNAs unravel their biological significance, we prepared a list of predicted targets for miR-135 using the TargetScan 7.2 program [40–42]. We found 280 conserved predicted targets across 8 species (human, mouse, rat, chimpanzee, rhesus, cow, dog, and opossum), highlighting the plausible evolutionary conserved functions of miR-135 (Fig. 1 and Table 1). To delineate miR-135-modulated, evolutionary conserved networks or pathways, we used the common list from the TargetScan program to reveal enriched pathways through Enrichr/DAVID analysis [41, 42]. Enrichr and DAVID analysis revealed pathways involving cGMP-PKG signaling, thyroid hormone signaling, Hippo signaling, insulin signaling, fluid shear stress, and atherosclerosis as the most enriched biological pathways. Notably, the TGFβ and FOXO signaling pathways were significantly enriched (*p*-value < 0.05) in both Enrichr and DAVID analyses. In addition to these pathways being dysregulated in cancer, several neurological processes such as dopaminergic synapse, axon guidance, and the synaptic vesicle cycle have also been shown to be affected by miR-135. Literature indicates an association between miR-135 and the TGFβ signaling pathway, as miR-135 binds to the 3’UTR of TGFBR1, TGFBR2, and SMAD3. MiR-135 targets TGFBR1, SMAD3, and CCND2, inhibiting granulosa cell growth and breast cancer progression, respectively [43]. Overexpression of miR-135b stimulates the proliferation and migration of colorectal and gastric cancer cells by binding to TGFBR2, suggesting an oncogenic role for miR-135 [44, 45]. MiR-135 also targets FOXO1 and exhibits oncogenic functions by promoting growth in melanoma and bladder cancer cells [46, 47].

**Table 1:** List of 280 conserved targets of miR-135 among eight species.

FOXN3	ST6GAL2	CALN1	BBX	ARHGEF4	POU2F3	AHCYL1	RIMS2	SPOCK1	VCAN
SLC24A2	PDE8B	DYRK1B	MEF2A	PPP6R3	PDE7B	WAPAL	SHISA6	GPM6B	ATG14
SMIM13	C7orf41	DNAJC16	YBX2	CASZ1	TEX2	KCTD1	PTPRD	CRAMP1L	BMPR2
KCNB1	MFHAS1	TAF4	PCNXL2	BMPER	BMPR1A	AREL1	BTAF1	CUX2	YTHDF3
KCNN3	GMFB	PHLDB2	PHOSPHO1	MSL2	ZBTB46	SLC8A1	RALGPS2	PVRL1	STRBP
SNTB2	PCDH9	BZW2	ZCCHC14	SLC5A7	KCNK12	FUT9	IGF2BP1	MED1	FRMD4A
LZTS1	JHDM1D	RPS6KB1	ETV3	NET1	CHD1	PHF17	ELK4	ZBED4	CCDC50
CPLX2	DKFZP779J2370	CCSAP	TRPC1	NDRG4	HIPK3	NCOA2	ZNF362	RP11-315D16.2	STAT6
PELI2	MYEF2	ZNF143	ATP2B2	SCN2B	CHST11	INHBA	GSK3B	NPAT	FTO
ANXA7	TNPO1	PPP1CC	DIP2C	FRMPD4	RARA	ZNF704	USP42	SYNCRIP	ZBTB44
NR3C2	SLC6A5	ACVR1B	WSCD2	TFAP2A	DGKH	DUSP5	NFXL1	LPGAT1	GABRB1
ELOVL6	MAPRE2	FOXN2	HMGXB3	SETD7	RP11-1055B8.7	KLHL28	ATP8A1	KIAA1324L	GATC
SSR1	ATP1B1	C6orf106	GRID2	DPF1	ZSWIM4	GABRB2	IRS2	GLYCTK	NUP153
TCF7L2	FKBP1A	MTDH	CADM3	CNIH1	ARHGEF2	ZBTB34	LRRN1	SLITRK6	KDM5B
KCNAB3	MYO9A	HMBOX1	CADM3	PDE7A	SIRT1	EXOC5	SETBP1	BCL9L	AL021546.6
C14orf166	PGGT1B	BTBD10	FCHO2	SGMS1	EFNB2	MTMR12	MYT1L	KCNJ6	TRAF4
ZRANB2	FOXO1	FBXO28	SIAH1	DCUN1D4	ARHGEF6	ROCK1	JOSD1	TBC1D30	HIC2
NEFM	TBX4	ELOVL2	PTK2	MEF2C	LDB2	NPTX1	RCOR1	PANK3	SPATA2
ELK3	SKOR1	VLDLR	PCYT1B	CHSY1	RIMS1	DAG1	ZNF652	LANCL3	RSBN1L
C6orf120	MOB1B	RGL1	KLF3	TRAPPC8	TRPM7	BACE1	PELI1	DDX3X	MEGF11
RASAL2	TOPORS	LATS2	EIF5A2	MED13	RAP2A	CBLB	LMTK2	SCN2A	THRA
ZNF385B	TSEN54	UBOX5	TET3	PAK7	SEC14L1	SLCO5A1	LMX1B	RBFOX2	HOXD9
ADCYAP1	JAKMIP2	KLF4	AEBP2	SYT1	KAT6B	DLGAP2	DLG2	SEMA6D	HNRNPA3
ADARB2	PRLR	SLC30A4	YWHAG	MAPK10	ZDHHC6	VAMP2	ZNF217	PLCB1	BRWD1
SP3	RALBP1	PPP2R5C	RORB	EBF1	WAC	QKI	RYBP	EPHA7	STK35
ZNF654	CCNG2	LRRFIP1	SNX18	C16orf52	FAXC	CCDC171	GGA1	USP31	COG5
ZNF131	MAN1A1	GATA3	NUCKS1	CSMD1	GGNBP2	ERBB4	CDYL2	ARHGAP32	AGPS
ENTPD4	RORA	CACNA1E	GRIK3	ATXN7L1	DTNA	EDEM3	CALML4	PRDM16	ABI2

The thyroid hormone signaling pathway affects miR-135, as levels of miR-135 were found to increase upon stimulation by thyroid hormones in HepG2 liver cancer cell lines [48]. Further studies suggest an association between miR-135 and the insulin signaling pathway [49]. Agarwal et al. show that miR-135a downregulates IRS2 and inhibits insulin signaling and glucose uptake in diabetic skeletal muscle [50–52]. Several reports highlight the involvement of miR-135 in atherosclerosis, as it modulates the proliferation and migration of vascular smooth muscle cells [45, 46, 51, 52]. Studies also suggest an association between miR-135 and the Hippo signaling pathway, as shown by a negative correlation between key components of the Hippo signaling pathway, such as LATS2, β-TRCP, NDR2, and LZTS1, and miR-135 [53]. Notably, miR-135 targets several genes in the Hippo signaling pathway, indicating stringent regulation of the pathway by miR-135.

## Brain tumor

The most common malignant brain tumor in adults is glioma, while non-malignant brain tumors include meningioma and pituitary tumors. Glioma, glioblastoma, and medulloblastoma exhibit mutations in DNA mismatch repair genes [5]. Gliomas are the most common primary brain tumors, arising from the glial cells or precursor cells of the central nervous system (CNS) [54]. Gliomas are phenotypically classified into two categories: (1) astrocytic, which includes astrocytoma and oligoastrocytoma, and (2) oligodendroglial, which includes oligodendroglioma and oligoastrocytoma. Ependymoma is a glial cell tumor that arises in the lining cells of the ventricular system of the CNS [55]. Based on WHO grading, gliomas can be classified from Grade 1 (least aggressive) to Grade 4 (most aggressive), and based on mutations in the isocitrate dehydrogenase gene, the tumor can be classified as IDH mutant or IDH non-mutant [56].

Glioblastoma (GBM) is the most common WHO Grade 4 malignant primary brain tumor that arises from the supporting cells of the central nervous system, known as glial cells. It is characterized by aggressive vascular proliferation, invasion, stem cell-like behavior, and chemoresistance, with patients showing less than a 5% survival rate and a median survival of less than 15 months [5, 57, 58]. GBM has a very poor prognosis and shows both inter- and intra-tumor heterogeneity [59]. Mitotically active GBMs are characterized by microvascular proliferation or necrosis and are diagnosed by genetic alterations such as epidermal growth factor receptor (EGFR) gene amplification, whole chromosome 7 gain, and whole chromosome ten loss [55, 60]. GBM also harbors many genetic and epigenetic mutations in genes such as IDH1, NOTCH, VEGF, and others [56].

Pediatric medulloblastoma (MB) is thought to originate from neuroepithelial stem cells in the cerebellum and occurs in the posterior cranial fossa [61, 62]. MB can be diagnosed at ages 6–8 years and in some individuals during adulthood [63]. The tumor is classified into four subgroups: WNT, SHH, Group 3, and Group 4 [61, 63]. The WNT subtype of MB arises from dorsal brainstem progenitor cells [64]. The SHH-activated subtype arises from the external granule neuron cells of the cerebellum. The SHH-activated MB is categorized based on mutated p53 into Medulloblastoma SHH-activated with wild-type p53 and Medulloblastoma SHH-activated with mutant p53 [65]. The non-WNT/non-SHH categories comprise Group 3 and Group 4 MBs. Group 3 tumors are overexpressed in infants and are located in the fourth ventricle, near the brainstem. Group 4 tumors are the most common type of MB [65, 66]. Enormous amplification of the Myc gene is a characteristic feature of MB [63].

## Glioblastoma organoids as in vitro model system

The main limitation of preclinical GBM models is the lack of a human microenvironment and the inability of tumor cell lines to replicate the human GBM characteristics. These challenges have been addressed through the development of three-dimensional (3D) models, such as tumor organoids [67], patient-derived xenografts (PDXs) [68], and genetic mouse models [69]. However, these models still have fundamental limitations. To investigate tumor initiation, progression, and drug testing, neoplastic cerebral organoids (neoCOR) were established from H9 hESC-derived cerebral organoids by introducing oncogene amplification and tumor suppressor gene mutations using Sleeping Beauty Transposon and CRISPR/Cas9 tools, respectively [70]. In addition to pluripotent stem cells, patient tissue samples can also be used to establish glioma organoids [59]. The glioma organoids derived from patient tumor tissues display significant tumor heterogeneity, gene expression patterns, 3D spatial organization, and tumor-microenvironment interactions, similar to the parental tumor. Key characteristics of glioma organoids, such as rapid generation, aggressive infiltration, intra- and inter-tumor heterogeneity, and efficient engraftment, make them useful for drug screening, toxicity assessment, and investigating the tumor microenvironment (Table 2) [71].

**Table 2:** Methods and limitations of generating brain tumor organoids.

Name of organoid	Disease	Initiator cell type	Purpose of study	Limitation	Reference
Patient-derived glioblastoma organoid	Glioblastoma	Surgically resected glioblastoma tissue	To biobanking the cells and to check the inter and intra-tumoral heterogeneity	Time span between resection and tissue processing is critical for the maximum reliability of organoid generation	[59]
Glioblastoma organoid	Glioblastoma	E2, R10 andG7 GBM cell line	To study radiation response and for drug modeling	Understanding of the complex pathway of VEGF signaling in GBM using the organoid is lacking	[161]
Glioblastoma- cerebral organoid co-culture model (GLICO)	Glioblastoma	U87-MG	To analyze the glioblastoma migration	The U87 cell line does not adequately represent the patient-derived tumor	[162]
Patient-derived glioblastoma organoid	Glioblastoma	Surgically resected glioblastoma tissue	To investigate drug resistance and treatment response	The time takes to establish the organoid is too long and the absence of TME, immune cells, and brain extracellular matrix is a failure of the method	[163]
Patient-derived glioblastoma organoid	Glioblastoma	Surgically resected glioblastoma tissue	For drug screening	The growth rate of gliosphere and PDO varies between patient samples because of the quality and proliferative nature of the tissue	[164]
Glioblastoma organoid	Glioblastoma	P3 Glioblastoma stem-like cells	For histological analysis of glioblastoma organoid	Inclusion quality of the invasive spheroid into the paraffin	[165]
Patient-derived glioblastoma organoid	Glioblastoma	U-251 MG cell line	Used as a precision medicine model and to study the invasion inhibition	The organoid lacks cellular heterogeneity because they made of only cancer cells	[166]
Low-grade glioma organoid	Low-grade glioma	LGG primary cell line from a patient	Biobanking and studying the parental tumor characteristics	The collected sample set was small and the study focused only on the method of preparation of organoid	[167]
GLICO	Glioblastoma	GSC,hESC	For GBM modeling and high throughput screening of drug		[168]
Cerebral organoid	Glioblastoma	hESC line H9	Glioblastoma modeling to observe tumor initiation		[169]
Human brain organoid	Glioblastoma	iPSC line 409b2	To study the invasion of GBM into the brain and transcriptional changes during the interaction between organoid and GBM		[170]
Glioma PDOXs (patient-derived orthotropic xenograft	Glioma	Glioma cell and GSC	For translational research and for studying personalized drug treatment	Molecular level interspecies difference, high cost, complex logistic, and low high-throughput nature	[171]
Laboratory engineered gliooblastoma like organoid (LEGO)	Glioblastoma	iPSCs	Found the property of glycerol lipid as a hallmark of GBM and provided a genotype-based drug reference map		[30]
GLICO	Glioblastoma	U-87 MG, SNB-19, UP-007 and MG	To study the growth and migration of GBM and the drug resistance of GBM		[172]
GBMO	Glioblastoma	Patient-derived GSCs	To study the intrinsic immune response of Glioma and the development of progenitors for GBM		[173]
Neoplastic cerebral organoid	Brain tumor	From healthy cerebral organoid	To study the aspects of tumor biology and the effect of drugs in the tumor model	Lacks of vasculature	[70]
Medulloblastoma organoid	Medulloblastoma	Patient-derived MB cell	Analysis of expression proteasome NPI-0052		[174]
Medulloblastoma organoid	Medulloblastoma	Patient-derived MB cell	For MB modeling and screening of drug		[175]
Medulloblastoma organoid	Medulloblastoma	hiPSCs	For linage study and drug screening	Differentiation efficiency of cerebellum organoids	[176]

However, one of the main limitations of GBM organoids is the lack of vasculature. As a result, features related to vasculature, such as glomeruloid microvascular proliferation, cannot currently be observed and studied in GBM. This limitation warrants further improvement of the protocol through the adoption of co-culture techniques (Table 2) [70].

## Role of miRNA in different brain tumors

### Glioblastoma

Recent studies indicate the presence of various miRNAs with oncogenic and pro-apoptotic roles [72, 73]. Specifically, 256 miRNAs, including miR-21 and miR-93, were found to be upregulated in glioblastoma and act as oncomiRs, while 95 miRNAs were downregulated and functioned as tumor suppressors, such as miR-7 and miR-34a [74]. MiR-124 also exhibits tumor-suppressive effects in GBM by inhibiting growth and promoting chemosensitivity through targeting AURKA [75]. MiRNA and epigenetic crosstalk have also been observed in GBM. For example, the knockdown of histone deacetylase 2 increases miR-3189 expression, which in turn represses GLUT3 expression and modulates glucose metabolism and tumor cell proliferation [76, 77]. MiR-128 suppresses the proliferation of glioma cells by targeting the transcription factor E2F3a and mitogenic kinases (Table 3) [78, 79]. Exosome-secreted miRNAs also modulate glioma progression. For instance, exosome-secreted miR-3591-3p facilitates macrophage polarization toward the M2 phenotype by targeting CBLB, promoting glioma progression, invasion, and migration (Table 3) [80].

**Table 3:** Brain tumor MiRNA’s function and targets.

Diseases	Type of miRNA expression	Target	Function	Reference
Glioblastoma	miR-138	CD44	Induce cell cycle arrest via p27 activation	[177]
	miR-7	EGFR, AKT, RAF1, FAK, and PI3K	Tumor suppression and inhibition of metastasis	[178, 179]
	miR-34a	MET, RTK, Notch, and CDK6	Tumor suppression	[180, 181]
	miR-3189	GLUT3	Tumor suppression by regulating glucose metabolism	[77]
	miR-93	Integrin β	Tumor cell proliferation and angiogenesis	[182]
	EV mediated miR-124	……	Inhibits M2 microglial polarization	[183]
	miR-128	E2F3a, mitogenic kinase	Suppress cell proliferation	[79]
	miR-21	PDCD4, IGFB3, Spry2, ANP32A, and SMARCA4	Oncogene	[184–188]
	miR-10b	BCL2L11, CDKN2A, CDKN1A, HOXD10, uPAR, and Rhoc/AKT	Promote cell progression, invasion, and metastasis	[189, 190]
	miR-3591-3p	CBLB and MAPK	Both as oncogene and suppressor	[80]
Medulloblastoma	miR-17-92	SHH/PTCH pathway, Cmyc	Development of the tumor	[88, 89, 191]
	miR-183-96-182	PI3K/AKT/Mtor	Apoptotic gene suppression	[92]
	miR-101-3p	FOXP4	Tumor suppression	[94]
	miR-423-5p	EZH2	Tumor suppression	[94]
	miR-137	KDM1A	Inhibits cell proliferation, migration, and invasion	[96]
	miR-326	E2F1	Tumorigenesis	[97]
	miR-1253	CDK6 and CD276	Suppress the tumorigenesis	[98]
Ependymoma	miR-10a and miR-10b	Chromatin modification genes		[103]
	miR-124	Metabolism regulating genes	Repress the cell communication	[103]
	miR-299 and miR-495-3p	PI3K/AKT	Up-regulated	[192]
	miR-29a/c	LAMA2	Down-regulated	[104]
	miR-124-3p	TP53INP1	Cell aggressiveness	[108]
	miR-485-5p	TGF-β	Down-regulated	[106]
	miR-15a	CYP11B1, KRT33B, RUNX1T1, and SIK1	Biomarker for poor prognostic	[110]
	miR-24-1	MAP3K4, MLANA, and SFRP5	promote tumor progression	[110]

**Fig. 2:**
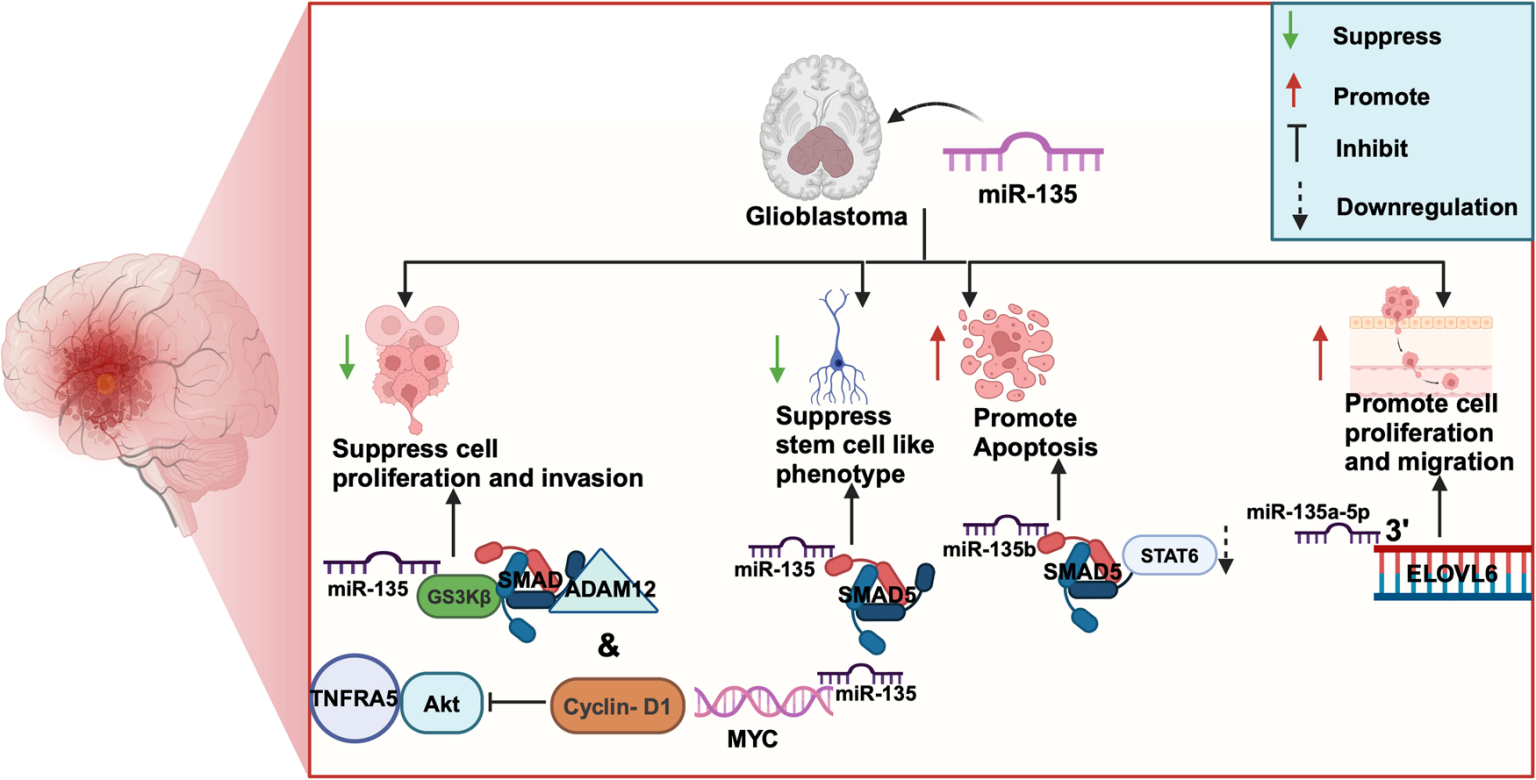
The schematic represents the roles of miR-135 in glioblastoma. The miR-135 suppresses cell proliferation and stem cell phenotype expression while the miRNA promotes apoptosis and migration.

MiR-135 is upregulated in many cancers, such as prostate and colorectal cancers, but downregulated in glioblastoma, and potentially acts as a tumor suppressor. In contrast, some studies suggest that miR-135 is upregulated in glioblastoma and promotes cell proliferation [81]. In glioma, the expression of miR-135b suppresses cell proliferation, invasion, and stem cell-like phenotypes by targeting SMAD5, ADAM12, and GSK3β (Fig. 2) [81]. MiR-135 also downregulates the expression of SMAD5 and STAT6, inducing apoptosis in glioma cells [82]. The novel niclosamide derivative NSC765689 upregulates miR-135b by modulating the GBM oncogenic signaling pathway genes such as GSK3β, β-catenin, STAT3, and CD44, and restoration of miR-135b function can reduce tumorigenesis and cancer recurrence [83]. MiR-135a-5p suppresses glioma proliferation by regulating the expression of c-myc and cyclin D1 via inhibition of the tumor necrosis factor receptor-associated factor 5 and AKT pathway [84]. The long non-coding RNA GACAT3 regulates the expression of NAMPT by sponging miR-135a and promotes glioma cell progression [85]. A recent study suggests that miR-135 plays an important role in lipid metabolism in glioblastoma cells. Both miR-135a-5p and miR-135b-5p bind to the 3’ UTR of ELOVL6 and regulate cell proliferation and migration in glioblastoma (Fig. 2) [86].

### Medulloblastoma (MB)

The miRNA expression profile of MB reveals 31 significant miRNAs that play key roles in the transcriptional repression of signaling pathways such as WNT, SHH, and MAPK [87]. The oncomiR-17–92 cluster is overexpressed in MB, where it is induced by N-myc and further activates the SHH signaling pathway, contributing to the development and progression of MB [88, 89]. Silencing of miR-9 through CpG island hypermethylation contributes to MB cell differentiation, cell cycle progression, and poor prognosis [90]. MiR-124 is downregulated in MB, and the restoration of its function inhibits cell proliferation, indicating the tumor-suppressive nature of this miRNA [91]. The miR-183-96-182 cluster is found to be upregulated in non-SHH MBs, while the expression of miR-182 is higher in metastatic MBs compared to non-metastatic MBs. Enhanced levels of miR-182 correlate with increased cell migration in vitro and metastatic dissemination in vivo in non-SHH MBs by regulating the PI3K/AKT/mTOR axis [92]. The intracellular ROS levels are regulated by miR-128, which targets the oncogene Bmi-1 and promotes senescence, suggesting a tumor-suppressive role for miR-128 [93]. Significant upregulation of miR-101-3p and miR-423-5p has been reported in plasma exosomes of MB patients. These exosomal miRNAs act as tumor suppressors by inhibiting growth, migration, and invasion of tumor cells, as well as elevating apoptosis via targeting the FOXP4 transcription factor and EZH2 histone methyl transferase [94]. The lncRNA HOTAIR enhances the growth, migration, and invasion of MB cells by stimulating YY1 expression through sponging miR-1/miR-206 [95]. MiR-137 is found to be downregulated in MB, and its overexpression inhibits cell proliferation, migration, and invasion while inducing apoptosis via targeting KDM1A [96]. MB tumorigenesis is promoted by the low expression of miR-326 and its host gene, β-arrestin1, which enhances the pro-survival function of E2F1 [97]. MiR-1253 shows tumor-suppressor activity by inhibiting CDK6 and CD276, promoting apoptosis, cell cycle arrest, and suppressing tumorigenesis in MBs (Table 3) [98].

**Fig. 3:**
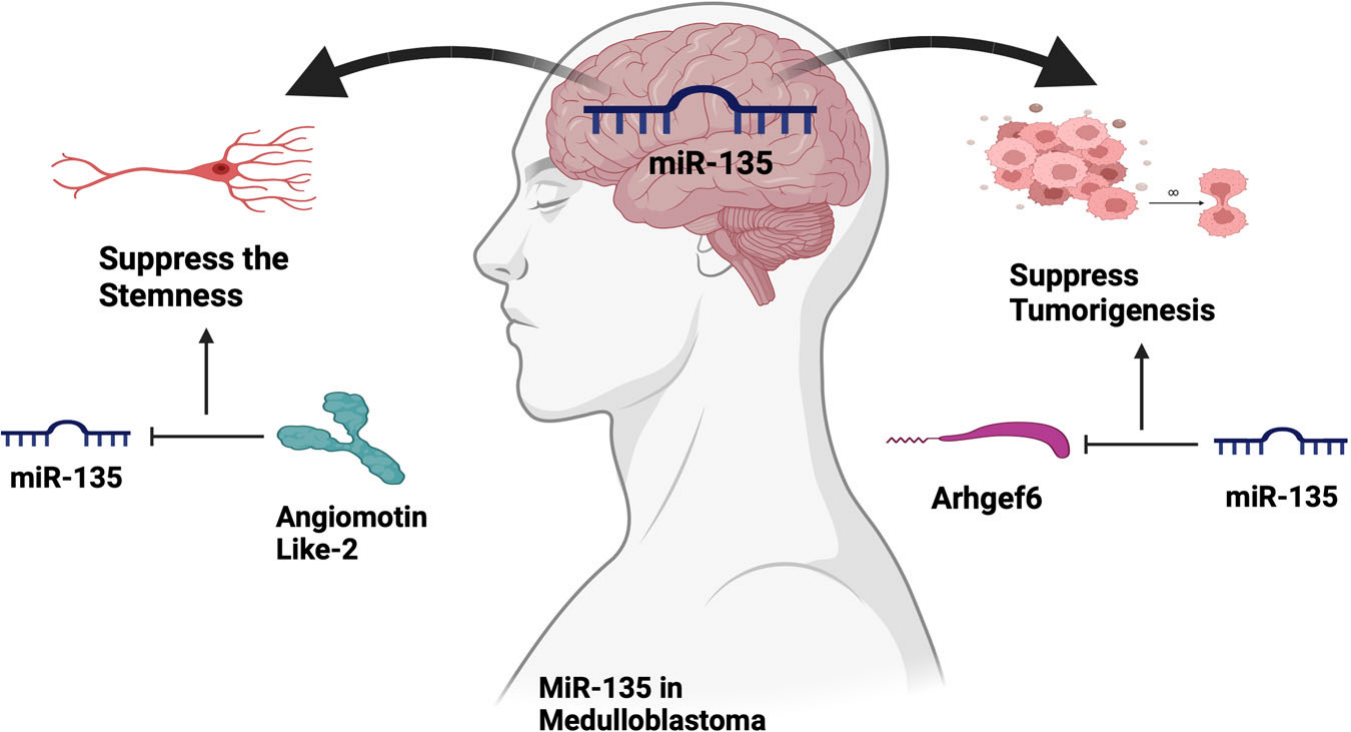
The schematic represents the potential roles of miR-135 in medulloblastoma. MiR-135 suppresses the stemness property and tumorigenesis.

MiR-135b is overexpressed along with its host gene LEMD1 in groups 3 and 4 of MB tumors [99]. A recent report also demonstrates the upregulation of miR-135a and miR-135b in brain tumor spheroid-forming cells and extracellular vesicles. Their inhibition reduces the stemness of cells by regulating angiomotin-like 2 (Fig. 3) [100]. In contrast, the downregulation of miR-135a suppresses the expression of Arhgef6 (alpha-PIX) and inhibits tumorigenesis in CSC-driven MBs (Fig. 3) [101].

### Ependymoma (EPN)

EPN exhibits overexpression of miR-10b and miR-29a, along with low expression of miR-10a [102]. In pediatric spinal ependymomas, upregulation of miR-10a and miR-10b leads to the targeting of chromatin modification genes [103]. Downregulation of miR-29a/c alters the expression of LAMA2 in EPN [104]. Recent reports have revealed the overexpression of miRNAs such as miR-34b-c, miR-200a-b, and miR-483, while several miRNAs, including miR-124a, miR-137, miR-138, miR-181d, miR-193b, miR-383, and miR-485-5p, are under expressed [105]. MiR-485-5p targets members of the TGF-β family [106]. The high expression of miR-203a in Grade III EPN serves as a prognostic and diagnostic marker, and its low expression is correlated with tumor recurrence [107]. High expression of miR-124-3p in EPN may correlate with poor progression-free survival in EPN patients [108]. MiR-124 represses cell communication and metabolism-regulating genes in EPN [103].

In EPN, most members of the miR-17-92, miR-106a-363, and miR-106b-25 clusters are highly expressed, especially miR-18a and miR-18b, which show the highest expression. Other miRNAs that show high expression in EPN include miR-19a, miR-92a, miR-106a, miR-93, miR-25, miR-17-5p, miR-20a, and miR-363 [109]. MiR-15a and miR-24-1 act as biomarkers for poor prognosis in childhood EPN. MiR-15a targets the 3’ UTR of CYP11B1, KRT33B, RUNX1T1, and SIK1, while miR-24-1 targets include MAP3K4, MLANA, and SFRP5. The interaction between miR-24-1 and its targets regulates the expression of the MAPK, NF-κB, and WNT signaling pathways, which promote tumor progression in EPN (Table 3) [110].

MiRNA profiling of Formalin-Fixed Paraffin-Embedded (FFPE) EPN samples and normal brain tissue revealed the overexpression of miR-135 along with miR-17-5p in EPN samples [106]. Another miRNA profiling performed on FFPE low-grade glioma (LGG), EPN, medulloblastoma (MED), and high-grade glioma (HGG) patient brain tumor tissues, including glioblastoma, medulloblastoma, and ependymoma, corroborated the overexpression of miR-135 in ependymoma tissue [102].

## Neurodegenerative diseases

### Alzheimer’s disease (AD)

AD is one of the top-ranked progressive, age-related neurodegenerative disorders, with dementia as its primary characteristic feature. The complete pathophysiology of AD remains unclear, but several theories have been proposed. Two theories have gained wider acceptance: one suggests the accumulation of beta-amyloid protein, where plaques consisting of extracellular amyloid beta (Aβ) proteins and neurofibrillary tangles (NFTs) composed of hyperphosphorylated tau protein are present [111]. The second theory supports the degeneration of acetylcholine-producing neurons in various parts of the brain, including the cerebral hemisphere, hippocampus, and pons, leading to progressive cognitive and memory deficits [112, 113]. Around 70% of all miRNAs are expressed in the brain alone. Several studies have shown that dysregulated miRNAs can contribute to the progression of AD [114].

**Fig. 4:**
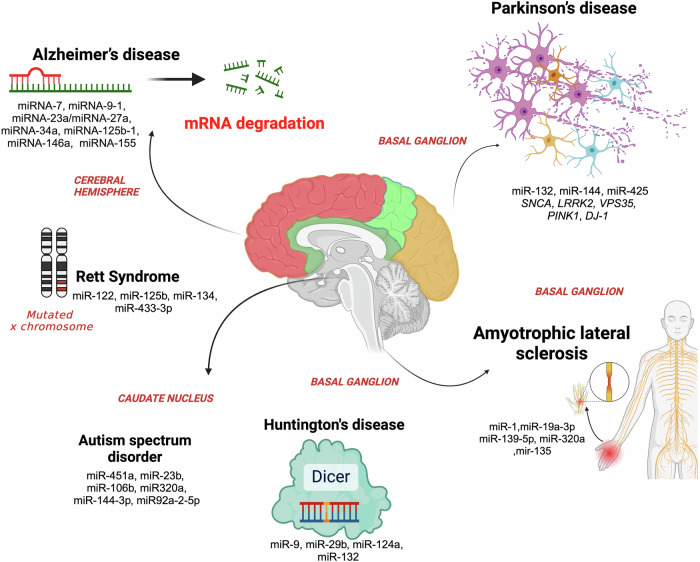
Dysregulation of miRNAs in neurological diseases. The schematic representation of prominentmiRNAs dysregulated in respective neurological diseases.

Various miRNAs involved in the pathophysiology and progression of AD have been found to be upregulated in the human temporal lobe neocortex. These include miR-7, miR-9-1, miR-23a/miR-27a, and miR-34a (Fig. 4) (Table 4) [115]. These upregulated miRNAs have the potential to bind to the 3’ UTR of their target mRNAs, which are involved in immune functions such as phagocytosis, innate immune response, inflammation, amyloid formation, and clearance. This binding leads to degradation or translational repression of their targets, contributing to the progression of AD [115]. Moreover, the levels of miR-4449 in cerebrospinal fluid (CSF) have emerged as a potential, highly accurate diagnostic tool for AD [116].

**Table 4:** Implication of miRNAs in different neurological diseases.

Disease	Type	miRNA upregulation	miRNA downregulation	Cells/tissue	Reference
Alzheimer’s disease	Serum circulating mi-RNAs	miRNA-7, miRNA-9-1, miRNA-23a/miRNA-27a, miRNA-34a, miRNA-125b-1, miRNA-146a, and miRNA-155	miR-132/212, miR-335–5p, miR-124, and miR-425–5p	Blood sample and cerebrospinal fluid	[115, 193]
Parkinson’s disease	Tissue specific mi-RNAs	miR-184, miR-153, and miR- 205	miR-7, miR-190, and miR-181	Human embryonic kidney cells	[193, 194]
Huntington’s disease	Tissue specific mi-RNAs	miR-196a	miR-128a	human embryonic kidney cells	[195]
Amyotrophic lateral sclerosis	Serum circulating mi-RNAs	miR-1, miR-19a-3p, miR-133b, miR-133a-3p, miR-144-5p, miR-192-3p, and miR-192-5p	miR-139-5p, miR-320a, miR-320b, miR-320c, miR-425-5p, and let-7d-3p	Blood sample	[196]
	Muscle specific mi-RNAs	miR-206	miR-1, miR-133b	Muscle biopsy	[132]
		miR-23a, miR-29b, miR-206 miR-455, and miR-31	–	Skeletal muscle biopsy	[197]
	Leukocyte specific mi-RNAs	miR-338-3p	miR-149, miR-328-5p, miR-451, miR-583, miR-638, miR-665, and miR-1275	Blood sample leukocyte	[198]
		let-7b, miR-9, miR-124a, miR-132, miR-206, miR-338, miR-451, miR-638, and miR-663a	–	Blood sample leukocyte	[199]
Rett syndrome	Tissue specific mi-RNAs	miR-29b, miR329, miR-199b, miR-382, miR-296, miR-221, and miR-92	miR-146a, miR-146b, miR-130, miR-122a, miR-342, and miR-409	Rett syndrome infected mice brain	[200]
Autism spectrum disorder	Serum circulating mi-RNAs	miR-106a/b and miR-23b	miR-150-5p and miR-451a	Blood and saliva	[144]

Researchers have further studied the levels of brain-specific miR-135 in both animal and human patient tissues. In an AD mouse model, the levels of miR-135a-5p were significantly decreased in excitatory hippocampal neurons. FOXD3-mediated suppression of miR-135a-5p facilitated synaptic and memory impairments in a tau-dependent manner [33]. MiR-135 levels were also found to be lower in the AD patient group, and it targets the BACE1 protein by binding to its 3’ UTR [117]. In contrast, another study showed increased levels of miR-135a in serum exosomes of patients with dementia of the Alzheimer’s type (DAT). Furthermore, this miRNA could also serve as a diagnostic marker and is useful for the early detection of AD [118].

### Parkinson’s disease (PD)

PD is the second most common progressive neurodegenerative disease after AD. It is characterized primarily by tremors and is further associated with rigidity, bradykinesia, and postural instability. Age and gender influence the severity of the disease [119]. Like AD, the pathophysiology of PD is still not fully understood, although a few genetic factors and neurological pathways have been identified. Recent findings have also highlighted the role of neurotransmitters, particularly dopamine, as a major cause of the disease. Dopamine reduction occurs due to the death of dopaminergic neurons in the substantia nigra region of the basal ganglia [120]. Since the basal ganglia are involved in motor control and neural connections from this region extend to other body parts, the degeneration of dopaminergic neurons in the basal ganglia leads to the spread of tremors [119].

Mutations in genes such as SNCA, LRRK2, VPS35, PINK1, DJ-1, and PARK2 have been found to be associated with the progression of PD in about 10% of affected cases. Additionally, several studies confirm the involvement of miRNAs in the pathophysiology of PD (Table 4). Dysregulation of miRNAs such as miR-34b, miR-218, and miR-221 has been found to contribute to the development of PD by interacting with the 3’ UTRs of genes such as DJ-1, PRKN, and SNCA [121]. DJ-1 plays a role in protecting cells from oxidative stress. When miRNAs interact with the 3’ UTR of DJ-1, they cause its degradation through the process of ubiquitination. A few miRNAs contribute to neuronal programmed cell death and autophagy, including miR-133b, miR-126, miR-132, miR-144, miR-425, and miR-124 (Fig. 4). Therefore, the dysregulated miRNAs identified in PD patients’ brain samples or circulating fluids could serve as biomarker tools for the diagnosis and prognosis of PD [122].

A study reported abnormally high expression of miR-135b-5p in the dopaminergic neurons of PD patients [31, 117]. Kefeng et al. observed that the downregulation of miR-135b-5p partially reversed the increased cell proliferation and reduced cell apoptosis, which were mediated by the knockdown of MALAT1. This suggests that miR-135b-5p plays a protective role in MPP+-stimulated PD cell models [123]. In another MPTP-induced mouse model of PD, miR-135a-5p suppresses rho-associated protein kinase 2 (ROCK2) by binding to its 3’ UTR and mediates the protective effects of hydrogen sulfide against PD symptoms in the mouse model [124]. Additionally, SHSY5Y cells treated with MPP+ exhibit symptoms of PD, and overexpression of miR-135b in these cells plays a protective role by downregulating GSK3β, promoting cell proliferation, repressing apoptosis, and reducing neuroinflammation [125]. Moreover, in PD patients, the upregulation of miR-135 is associated with the downregulation of key transcription factors (TFs) such as FOXA1 and NR3C1. These TFs are involved in the regulation of axonal transport, cell adhesion, and survival in PD [126].

### Amyotrophic lateral sclerosis (ALS)

ALS, also known as “Lou Gehrig’s disease,” was initially considered a motor neuron disease. However, later research classified ALS as a neurodegenerative disease. It is an age-related, progressive disorder characterized by the loss of motor control in the upper and lower limbs, leading to difficulties in holding objects and walking or running [127]. Over time, the degeneration of motor neurons in the brainstem, anterior horns of the spinal cord, and motor cortex contributes to the severity of the disease [128].

ALS is classified into two primary forms: sporadic and familial. Sporadic ALS develops without any known family history and accounts for the majority of cases. In contrast, familial ALS, which represents around 5–10% of cases, is inherited and tends to run in families. Genetic mutations, such as those in the SOD1, C9orf72, TARDBP, and FUS genes, are associated with familial ALS, disrupting motor neuron function and leading to progressive muscle weakness [129]. The progression of ALS leads to partial paralysis, followed by complete paralysis, and ultimately results in death. The disease’s spread is not limited to the limbs; in most cases, the loss of function of the respiratory muscles occurs, leading to a decrease in the body’s ability to exchange gases. The neurophysiology of ALS is not fully understood, and there is no cure available to date [130].

Several miRNAs have been found to be dysregulated in ALS (Fig. 4) (Table 4). A study on familial and sporadic ALS patients reported consistent downregulation of miR-1234-3p and miR-1825 in sporadic ALS. These two miRNAs were downregulated only in ALS and not in patients with AD or Huntington’s disease, as confirmed by qRT-PCR [131].

In ALS, the expression level of miR-135a has been found to be downregulated. This downregulation of miR-135a is often correlated with hypertrophy, physical inactivity, fiber loss, and atrophy, which contribute to the development of ALS [132].

### Huntington’s disease (HD)

HD is a neurodegenerative condition caused by a mutation in the HTT gene on chromosome 4. This genetic alteration results in an abnormal elongation of the polyglutamine tract in the huntingtin protein. As a result, the mutant protein aggregates within neurons, leading to disruptions in cellular functions and proteostasis [133]. These pathological mechanisms ultimately lead to progressive neuronal dysfunction and death, giving rise to the characteristic motor, cognitive, and psychiatric symptoms associated with HD [134].

HD follows a dominant pattern of inheritance and is caused by a mutation in the gene, resulting in the accumulation of unstable polyglutamine repeats, specifically the trinucleotide “CAG” (cytosine, adenine, and guanine). Telenius and colleagues analyzed the number of CAG repeats in HD patient samples and demonstrated that a repeat number above 30 has a propensity to develop severe HD. Moreover, a patient with a CAG repeat number of 47 produced offspring with 121 polyglutamine repeats, increasing the likelihood of developing severe HD [135].

Loss of the Dicer gene is often associated with neurological diseases, and Huntington’s disease is one such example where the loss of Dicer-1 function contributes to the initiation and progression of the disease (Table 4). In HD, dysregulated levels of miRNAs such as miR-9, miR-29b, miR-124a, and miR-132, along with Dicer and Drosha, have been observed, suggesting that deregulated miRNA biogenesis contributes to HD progression (Fig. 4) [136]. Studies also show that some of the dysregulated miRNAs leading to HD are common across several species, including mice, monkeys, and humans, confirming an evolutionary relationship. Similar to HD patients, downregulation of miR-128a and upregulation of miR-196a has been observed in a transgenic monkey model of HD [137].

MiR-135 levels were significantly increased in pre-symptomatic HD patients, and these levels were positively correlated with the risk of disease onset [138]. In contrast, the expression levels of miR-135b were downregulated in animal models of HD [139]. This was corroborated in a study that reported reduced levels of miR-135b in the cortex and hippocampus of the brain at 12 weeks in an HD mouse model [140]. However, significant alterations in miR-135b levels were not detected with increasing disease severity in the Brodmann area 4 (BA4) cortex from postmortem human brain samples [141].

## Neurodevelopmental disorders

### Autism spectrum disorder (ASD)

ASD is a neurodevelopmental disorder that manifests as deficits in language, social interaction, and the presence of stereotypical repetitive behaviors. The condition is characterized by behavioral and psychological complications in neonates and children over the age of 2. The exact cause of the disorder remains unclear, resulting in the lack of therapeutic treatments [142]. Epidemiological data show that the prevalence of ASD is 1 in every 68 children in the United States and 1 in 100 children in India, with a rapid increase in the number of affected children worldwide [76]. Identifying ASD is challenging immediately after birth, but around the age of 2, parents of affected children often begin to notice symptoms, which raise concerns about autism. Symptoms of autism include delayed speech, motor skill difficulties, challenges in cognition, and learning, impulsive and inattentive behavior, and unusual eating and sleeping patterns. Moreover, limited behavioral therapy coupled with antipsychotic drugs provides relief to the patients [143].

ASD is associated with altered expression of multiple miRNAs, including miR-451a, miR-23b, miR-106b, miR-320a, miR-144-3p, miR-92a-2-5p, miR-150-5p, and miR-486-3p (Table 4). Among these, miR-451a has been found to be most closely associated with the progression and worsening of the disease (Fig. 4). These miRNAs also act as potential biomarkers that can aid in diagnosis. Profiling of body fluids, such as blood and saliva, reveals the complexity and severity of ASD and has been used to isolate miR-451a [144]. Since the genetic cause of ASD is not yet well understood, it seems unlikely that these miRNAs alone are responsible for causing ASD.

A study using the Slc6a4-knockout mouse model, which lacks the serotonin transporter required in the synaptic cleft of presynaptic neurons, revealed upregulation of miR-135a-5p. Further analysis indicated that miR-135a-5p targets important ASD-linked genes, such as CHD7 and CLCN3 [145]. The roles of these genes in neuronal development and neuron adhesion support their involvement in the progression of ASD. MiRNA profiling of the brains of 3-week-old maternal immune activation (MIA) mice showed downregulation of miR-135a-5p upon immune activation, with an inverse relationship to SNHG11 and FAM228B, both of which perform brain-specific functions. This finding also supports the idea that miRNA dysfunction mediates DNA methylation, thereby increasing vulnerability to ASD [146].

### MiRNA-based drugs in clinical trials for brain tumors and neurological disorders

MiRNAs have been employed as mimics, siRNAs, inhibitors, and antisense oligonucleotides for the therapeutic treatment of brain tumors and neurological diseases. MiRNA mimics are artificially synthesized double-stranded RNA molecules that harbor the same sequence as mature and passenger miRNA strands, where one strand acts as the “guide strand” and the other as the “passenger strand.” These miRNA mimics are recognized by the cytoplasmic miRNA biogenesis machinery, and the guide strand is incorporated into the RISC, followed by degradation of the passenger strand [147]. To increase cellular uptake and enhance the stability and integration of the guide strand into the RISC, various proprietary chemical modifications have been applied, such as the fusion of cholesterol with the passenger strand and fluorescein isothiocyanate (FITC) with the active strand [147, 148]. Such modifications have already entered clinical trials. For instance, MRX34, a mimic of miR-34a conjugated with liposomes, has entered Phase-I clinical trials for the treatment of primary and metastatic liver cancer (NCT01829971).

Oncogenic or disease-progressing miRNAs can be preferentially inhibited by miRNA inhibitors. Several proprietary chemical modifications, such as 2’-*O*-methyl, 2’-fluoro, 2’-*O*-methoxyethyl, 2’,4’-methylene, locked nucleic acid, and morpholino, along with phosphorothioate linkages, have been applied to these inhibitors to increase their stability and efficiency. This category includes anti-miRNA oligonucleotides (AMOs), which can inhibit either the whole miRNA sequence or the seed sequence [147, 149]. A clinical trial in the U.S. uses AMT-130 for the delivery of precursor miR-451a using an adeno-associated viral vector serotype 5 (AAV5) to prevent HD progression. miR-451a acts not only as a tumor suppressor but also suppresses HTT in HD (NCT04120493) [150]. Similarly, another ongoing clinical trial (NCT06100276) evaluates the safety, tolerability, and efficacy of gene therapy AMT-162 in ALS patients with an SOD1 mutation. AMT-162 encodes an artificial miRNA that targets the SOD1 gene and uses an adeno-associated viral vector serotype 10 for its delivery [150]. Additionally, miRNA sponges have been developed, which can inhibit single miRNAs as well as groups of miRNAs. Mechanistically, these sponges contain several binding sites for a single miRNA or a group of miRNAs and possess strong promoters for their expression [151].

Despite these consistent efforts, several major limitations hinder the commercial development of miRNA-based therapeutics. One major challenge is their safe and efficient delivery in a tissue-specific manner. Although adeno-associated viruses have been used for specific delivery, issues such as off-target effects, efficiency, and toxicity still persist [152]. Another problem is that the cell membrane may repel hydrophilic and negatively charged oligonucleotides. Researchers have used polymer-based carriers, lipid nanoparticles, and other chemical modifications of miRNAs to address this challenge [153]. Another significant obstacle is selecting the ideal miRNA for delivery, as a single miRNA can target thousands of genes. Furthermore, cellular conditions such as inflammation and hypoxia might alter miRNA expression. Several pre-clinical studies have shown that a particular miRNA may act as a tumor suppressor in one tumor and as an oncogenic miRNA in another. To address this ambiguity, a computational database has been developed to design artificial miRNAs that can target multiple genes at several sites, similar to siRNA [154]. Currently, oral formulations for miRNA-based therapy do not exist, even though the oral route is the most well-tolerated and preferred method of drug delivery [155]. Several roadblocks remain before miRNA-based therapies can be made available to patients [148, 150].

Several pre-clinical studies suggest that drugs, including melatonin, enhance the expression of miR-135 to treat various disease conditions [156]. However, other drugs, such as Morin, inhibit the expression of miR-135 to alleviate different abnormalities [157, 158]. Other neuro-mechanistic studies point to miR-135’s role as an endogenous antidepressant in depression, epilepsy, and memory deficits [36, 159, 160]. In summary, miR-135 has emerged as a therapeutic target that can be further explored in clinical trials for the development of novel drugs for the treatment of neurological diseases.

## Conclusion

This review elucidates the role of miRNAs in brain tumorigenesis, neurodegenerative diseases, and neurodevelopmental disorders, with a special emphasis on the brain-specific miRNA-135. MiR-135 plays a major role in brain tumorigenesis and neurological diseases. MiR-135 can act as both an oncogene and a tumor suppressor, though its low expression in major brain tumors such as glioblastoma and medulloblastoma suggests its tumor-suppressor ability. The tissue-specific context may influence the altered expression of miR-135 in different cancers. MiR-135 downregulates several targets involved in signaling pathways, thereby suppressing growth, proliferation, and tumor recurrence in glioblastoma and medulloblastoma. The miR-135-mediated regulation of several targets highlights the complexity of the associated signaling pathways. MiR-135 is reported to be downregulated in AD and ALS but upregulated in PD and HD, while increasing the vulnerability to autism. These findings suggest distinctive modes of regulation of this miRNA, potentially involving RNA-binding proteins, epigenetic factors, or transcription factors. MiR-135 not only acts as a diagnostic marker for several neurodegenerative diseases but also serves as a target for various drugs. Our enrichment analysis using the targets of miR-135 suggests that this miRNA participates in several signaling pathways, such as the cGMP-PKG pathway and thyroid hormone signaling, providing new insights into the mechanistic action of miR-135. Although the enriched signaling pathways have not yet been thoroughly assessed in neurological diseases, this warrants further investigation for the therapeutic development of miR-135-based modalities. The widespread role and involvement of miR-135 in various brain-associated diseases and its interaction with other signaling pathways highlight its significant clinical relevance for the development of therapeutic strategies aimed at ameliorating brain tumors and neurological diseases.

## Data Availability

All data generated or analyzed during this study are included in this published article.
